# Resolvin E1 Promotes Bone Preservation Under Inflammatory Conditions

**DOI:** 10.3389/fimmu.2018.01300

**Published:** 2018-06-12

**Authors:** Karim El Kholy, Marcelo Freire, Tsute Chen, Thomas E. Van Dyke

**Affiliations:** ^1^The Forsyth Institute, Cambridge, MA, United States; ^2^School of Dental Medicine, Harvard University, Cambridge, MA, United States; ^3^School of Dental Medicine, University of Bern, Bern, Switzerland; ^4^Faculty of Dentistry, McGill University, Montreal, QC, Canada; ^5^J Craig Venter Institute, La Jolla, MA, United States

**Keywords:** resolvin E1, tissue regeneration, bone, resolution, bone metabolism, inflammatory diseases

## Abstract

Resolvins are endogenous lipid mediators derived from omega-3 fatty acids. Resolvin E1 (RvE1), derived from eicosapentaenoic acid (EPA), modulates osteoclasts and immune cells in periodontal disease models. The direct role of RvE1 in bone remodeling is not well understood. The objective of this study was to determine the impact of RvE1 on bone remodeling under inflammatory conditions. Our working hypothesis is that RvE1 downregulates bone resorption through direct actions on both osteoblast and osteoclast function in inflammatory osteoclastogenesis. A tumor necrosis factor-α induced local calvarial osteolysis model with or without the systemic administration of RvE1 was used. To evaluate osteoclastogenesis and NFκB signaling pathway activity, murine bone tissue was evaluated by Micro CT (μCT) analysis, TRAP staining, and immunofluorescence analysis. Mechanistically, to evaluate the direct role of RvE1 impacting bone cells, primary calvarial mouse osteoblasts were stimulated with interleukin (IL)-6 (10 ng/ml) and IL-6 receptor (10 ng/ml) and simultaneously incubated with or without RvE1 (100 nM). Expression of receptor activator of NFκB ligand (RANKL) and osteoprotegerin (OPG) was measured by ELISA. RNA sequencing (RNA-Seq) and differential expression analysis was performed to determine signaling pathways impacted by RvE1. The systemic administration of RvE1 reduced calvarial bone resorption as determined by µCT. Histologic analysis of calvaria revealed that osteoclastogenesis was reduced as determined by number and size of osteoclasts in TRAP-stained sections (*p* < 0.05). Immunofluorescence staining of calvarial sections revealed that RvE1 reduced RANKL secretion by 25% (*p* < 0.05). Stimulation of osteoblasts with IL-6 increased RANKL production by 30% changing the RANKL/OPG to favor osteoclast activation and bone resorption. The ratio changes were reversed by 100 nM RvE1. RvE1 decreased the production of RANKL maintaining an RANKL/OPG more favorable for bone formation. RNA-Seq and transcriptomic pipeline analysis revealed that RvE1 significantly downregulates osteoclast differentiation mediated by differential regulation of NFκB and PI3K–AKT pathways. RvE1 reduces inflammatory bone resorption. This action is mediated, at least in part, by direct actions on bone cells promoting a favorable RANKL/OPG ratio. Mediators of resolution in innate immunity also directly regulate bone cell gene expression that is modulated by RvE1 through at least 14 specific genes in this mouse model.

## Introduction

The niche mediators and cells shaping metabolic control of bone tissue tightly regulate bone-remodeling circuitry. The coupling activities between mesenchymal origin bone forming cells, osteoblasts, with the myeloid origin bone resorbing cells, osteoclasts, are modulated by receptor activator of NFκB ligand (RANKL) and osteoprotegerin (OPG) biological actions (1, 2).

Inflammatory cytokines produced by osteoblasts and osteoclasts, including interleukin (IL)-1, IL-6, and tumor necrosis factor-α (TNF-α), activate the bone microenvironment, boosting expression of RANK and bone resorption phenotype (3). In chronic inflammatory conditions including periodontitis and rheumatoid arthritis, persistent chronic inflammation leads to loss of bone mass and volume, and is consistently accompanied by an increase in local expression of RANKL (4–8). Long and craniofacial bones are targets of unresolved inflammation and clinical consequences affect populations worldwide. An estimated 47% of the US population suffers from moderate to severe periodontal disease (9–11). According to the United Nations and WHO, musculoskeletal conditions (rheumatoid arthritis, osteoporosis, and osteoarthritis) are a major burden financially and socially on individuals, health systems, and social care systems (12). Thus, understanding activation and resolution (termination) of inflammation has potential impact in bone metabolism and human health.

Immune cells are regulated by specialized pro-resolving mediators (SPMs) (13–15), which were first characterized by unbiased systems approach to study acute inflammation and self-resolving inflammatory exudates (16, 17). A new genus of molecules was identified: lipoxins, resolvins, maresins, and protectins (collectively, SPMs) (18–20). Temporal lipoxygenase class-switching results in the emergence of lipoxins, derived from arachidonic acid, and resolvins, maresins, and protectins, derived from omega-3 fatty acids that drive resolution of inflammation and clearance of inflammatory lesions (15, 21). Inflammation resolution is not a passive mechanism resulting from decay of pro-inflammatory cytokines; instead, SPMs are *active* biochemical signaling molecules (14, 15, 22, 23) promoting debris and apoptotic cell clearance through lymphatics and a return to homeostasis. Resolvin E1 (RvE1) is derived from eicosapentaenoic acid (EPA), and along with the other mediators has been shown to have a modulatory role in inflammation-associated models of human diseases (24–28), including arthritis (29), colitis (30), peritonitis (14, 31), asthma (19, 24, 32), dermatitis (33), infantile eczema (34), diabetic wounds (35, 36), and retinopathies (37, 38).

The purpose of this study was to determine the mechanism of RvE1 actions in the prevention of bone resorption on a subcellular level. To build on our previous experiments, we used an *in vivo* model of bone resorption, rather than an artificial *in vitro* assay (the pit assay of dentin slices) (39) to characterize the RANKL/OPG axis *in situ* and at the molecular level. Specifically, the transcriptome of the osteoblast in inflammation was assessed with and without treatment with RvE1.

## Materials and Methods

### Animals

All experiments in this study were reviewed and approved by the Institutional Animal Care and Use Committee (IACUC) of the Forsyth Institute. Eighteen 5- to 6-week-old C57BL/6 mice (Charles River Laboratories, New York, NY, USA) were used for each experiment.

### Osteolysis Model

Mice were divided into three groups (control group, receiving PBS, calvarial injections: *n* = 6, TNF-α group, receiving TNF-α calvarial injections: *n* = 6, TNF-α + RvE1 group, receiving TNF-α calvarial injections + RvE1 intraperitoneal injections: *n* = 6). Mice were sedated with isoflurane 2–3% before being anesthetized with ketamine (80 mg/kg intraperitoneally) and xylazine (16 mg/kg, intraperitoneally). Daily supracalvarial injections of 100 µl of PBS or TNF-α (2 μg/100 μl) with or without daily intraperitoneal injections of 50 ng of RvE1 were performed for 7 days in six mice per group. Mice were sacrificed at the end of the experimental period (day 8), and all calvariae were dissected immediately after sacrifice. One calvarium from each group was defleshed with dermestid beetles for 24 h and processed for photographic imaging. The other five calvariae in each group were fixed overnight with 4% paraformaldehyde at 4°C.

Recombinant mouse TNF-α was purchased from R&D systems (Minneapolis, MN, USA). RvE1 (C_20_H_30_O_5_, molecular weight: 350.5, purity >97%, λmax: 272 nm) was purchased from Cayman Chemical (Ann Arbor, MI, USA). The RvE1 provided by Cayman was prepared by stereospecific total synthesis guided by the published structure described by Arita et al. (30). RANKL antibody conjugated with Alexa Fluorochrome 488, and OPG and IL-6 antibodies both conjugated with Dylight fluorochrome 550 were purchased from Novus Biologicals (Littleton, CO, USA). Specimens were demineralized in 10% EDTA for 4 days at 4°C. To count osteoclast number, the sections were stained for TRAP activity, and counterstained with hematoxylin. Sections for immunofluorescence were deparaffinized and incubated for 1 h with a mix of primary antibodies at designated concentrations. Slides were then viewed under fluorescent light and images were taken using Zeiss software.

### Osteoblast Isolation

Neonatal mice calvarial osteoblasts were isolated following the protocol of Wong and Cohn (40) from mouse litters (7–8 mice, 2–4 days old) by sequential incubation in collagenase type 2 solution (Worthington). Isolated primary cells were grown in two 75 ml flasks and after reaching 90% confluence were passaged into six-well plates at a density of 6 × 10^4^ cells/well. Medium was supplemented with vitamin D_3_ and ascorbic acid and was changed every other day for 10 days (41).

### RNA Isolation

Total RNA was isolated from murine osteoblasts with TriZol (Life Technologies, Carlsbad, CA, USA) (42), and purity was confirmed using a NanoDrop ND-1000 spectrophotometer (Thermo Scientific). RNA was stored for later use at −80°C in RNAlater. RNA was then reverse transcribed using a High Capacity cDNA Reverse Transcription Kit (Applied Biosystems). mRNA expression levels were quantified by real-time PCR using SYBR Select Master Mix (Applied Biosystems) on a Light Cycler 480 (Roche Diagnostics).

### RNA Sequencing (RNA-Seq)

Cells were collected immediately after the experiment was completed and processed for RNA-Seq on the Illumina Basespace platform. Total RNA was extracted from osteoblasts using an RNeasy Mini kit (Qiagen, Valencia, CA, USA). Raw read counts per gene were mapped to corresponding human homologs, using homology information from the Mouse Genome Informatics database (The Jackson Laboratory). The iPathwayGuide was used to score the biological pathways using impact analysis (43, 44). Impact analysis uses two values to measure magnitude of pathway being examined (1) the overrepresentation of differentially expressed genes in a pathway and (2) the perturbation of that pathway computed by propagating the measured expression changes across the pathway topology. These aspects are captured independent probability values; pORA, representing the probability of obtaining a number of differentially expressed genes on the given pathway greater or equal to chance observations; pAcc, represents the *p*-value obtained from total perturbation accumulation. Combining pORA and pAcc is done to produce a unique global *p* value using Fisher’s method, which is then corrected for multiple comparisons using the false discovery rate method.

### Immunofluorescence

Calvarial sections were incubated with anti-Fc receptor (BD) blocking antibody (5 µg/ml × 10^6^ cells, 15 min) and then labeled with anti-human RANKL Alexa Fluor 488-conjugated antibody (10 µg/ml × 10^6^ cells, 1 h at RT) or OPG PE-conjugated antibody isotype control, R&D System. Expression levels of the proteins were monitored by microscopy and analyzed with NIH Image J.

### ELISA

Cells were cultured for 10 days as described above and then stimulated with 10 ng/ml IL-6 and 10 ng/ml soluble IL-6 receptor (sl-IL-6R, Antigenix) with or without 100 nM RvE1, and incubated for 48 h. Supernatants were collected, centrifuged at 4,000 × *g* for 10 min at 4°C, and frozen at −80°C until assayed. RANKL and OPG ELISA kits were purchased from R&D Systems (Minneapolis, MN, USA).

### Micro CT

Samples were placed in a standardized sample holder and scanned using high-resolution Micro CT (μCT) (Scanco Medical, Brüttisellen, Sweden) at a spatial resolution of 18.676 µm (voxel dimension) with 1,536 × 1,536-pixel matrices. The two-dimensional image data were stored in Digital Imaging and Communications in Medicine format and transferred to a computer for three-dimensional reconstruction and analyses.

Murine calvarial regions were cropped from consecutive slice images as volume of interest (VOI) using Amira software (VSG|FEI Visualization Sciences Group, Burlington, MA, USA). The volume of new bone was measured within a digital VOI according to teeth dimensions. Volumetric analysis was evaluated by global thresholding procedures (bone tissue = 3,409–10,913). Total bone volume (BV) is presented as mean and SD of voxel units calculated by Amira (45). Statistical significance was determined by analysis of variance (ANOVA) and multiple comparisons were corrected by the Holm–Sidak method (significance at *p* < 0.05).

### Statistics

Comparisons between the three *in vivo* groups and the four *in vitro* groups were analyzed by one-way ANOVA followed by *post hoc* analysis (with Bonferroni adjustment in the pairwise *t*-test function). All values were expressed as mean SEM. *p*-Value lower than 0.05 was considered statistically significant.

## Results

### RvE1 Promotes *In Vivo* Bone Regeneration

**Figure 1:**
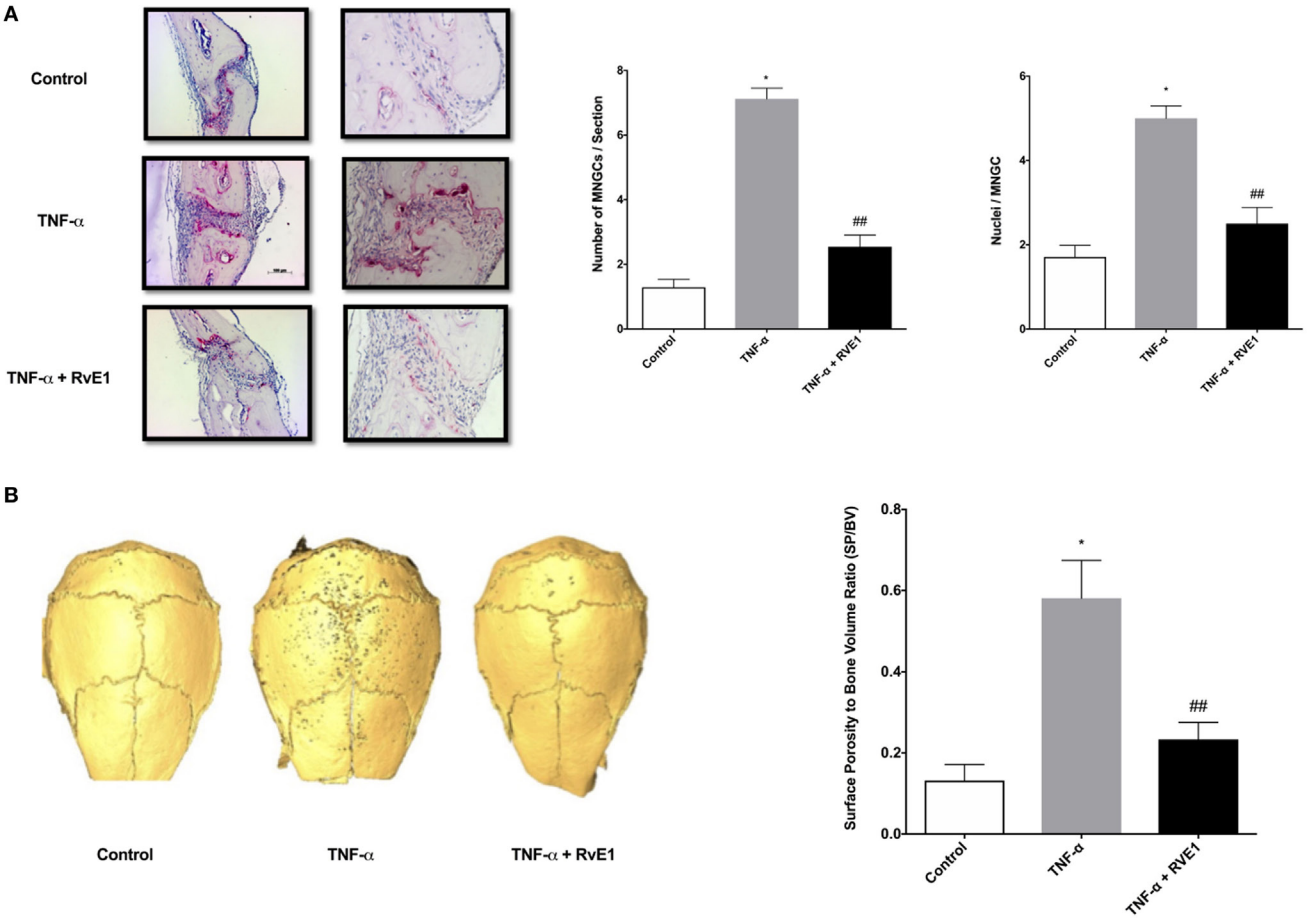
**(A)** RvE1 reduces osteoclastogenesis triggered by TNF-α injections. TRAP-positive multinucleated giant cells (MNGCs) were counted in each section and the mean number of nuclei/cell/section was calculated by dividing the number of nuclei by the number of MNGCs found in each section. Statistically significant increases in numbers of MNGCs and nuclei/MNGC/section were measured in sections from samples receiving supracalvarial TNF-α injections when compared to samples receiving PBS injections (*p* < 0.05). Statistically significant decrease in both values was found in samples from animals treated with daily injections of RvE1 when compared to the TNF-α only group (*p* < 0.05). **(B)** Protective role of resolvin E1 (RvE1) in murine calvariae. Significant increases in the surface porosity (SP)/bone volume (BV) ratio was noted in specimens from the tumor necrosis factor-α (TNF-α) group when compared to the negative control and RvE1 treatment group (*n* = 3, *p* < 0.05). Reconstruction was performed using Amira 3D software. BV and SP measurements were calculated using the same software. SP/BV measurements were calculated to standardize the surface area being examined for porosity. Results are expression of the mean SP/BV ratio. *p < 0.05 vs. control. ^##^*p* < 0.05 vs. active control.

To investigate if daily TNF-α injections induced calvarial osteolysis in mice, surface porosity (SP) and BV measurements were conducted by μCT using Amira 3D software to measure and quantify peaks and valleys on calvarial surfaces. The ratio of SP to BV was measured to standardize the BV being tested for SP. A significant increase in SP/BV ratio was observed in specimens from the TNF-α group compared to both negative control and RvE1 treatment groups (*p* < 0.03) (Figure 1A).

### RvE1 Reduces Osteoclastogenesis Triggered by TNF-α Injections

The number of TRAP-stained multinucleated giant cells (MNGCs) in the suture area in each section, as well as the number of nuclei per MNGC found in each section was analyzed (Figure 1B). The adjusted mean number of nuclei/cell/section was calculated by dividing the number of nuclei by the number of MNGCs found in each section. A statistically significant increase in number of MNGCs and nuclei/MNGC was observed in sections from samples receiving TNF-α injections compared to samples receiving PBS injections (*p* < 0.05). A statistically significant decrease was observed in samples from animals in the RvE1 treatment group compared to the TNF-α group (*p* < 0.05).

### RvE1 Modulates *In Situ* Expression of OPG and RANKL

**Figure 2:**
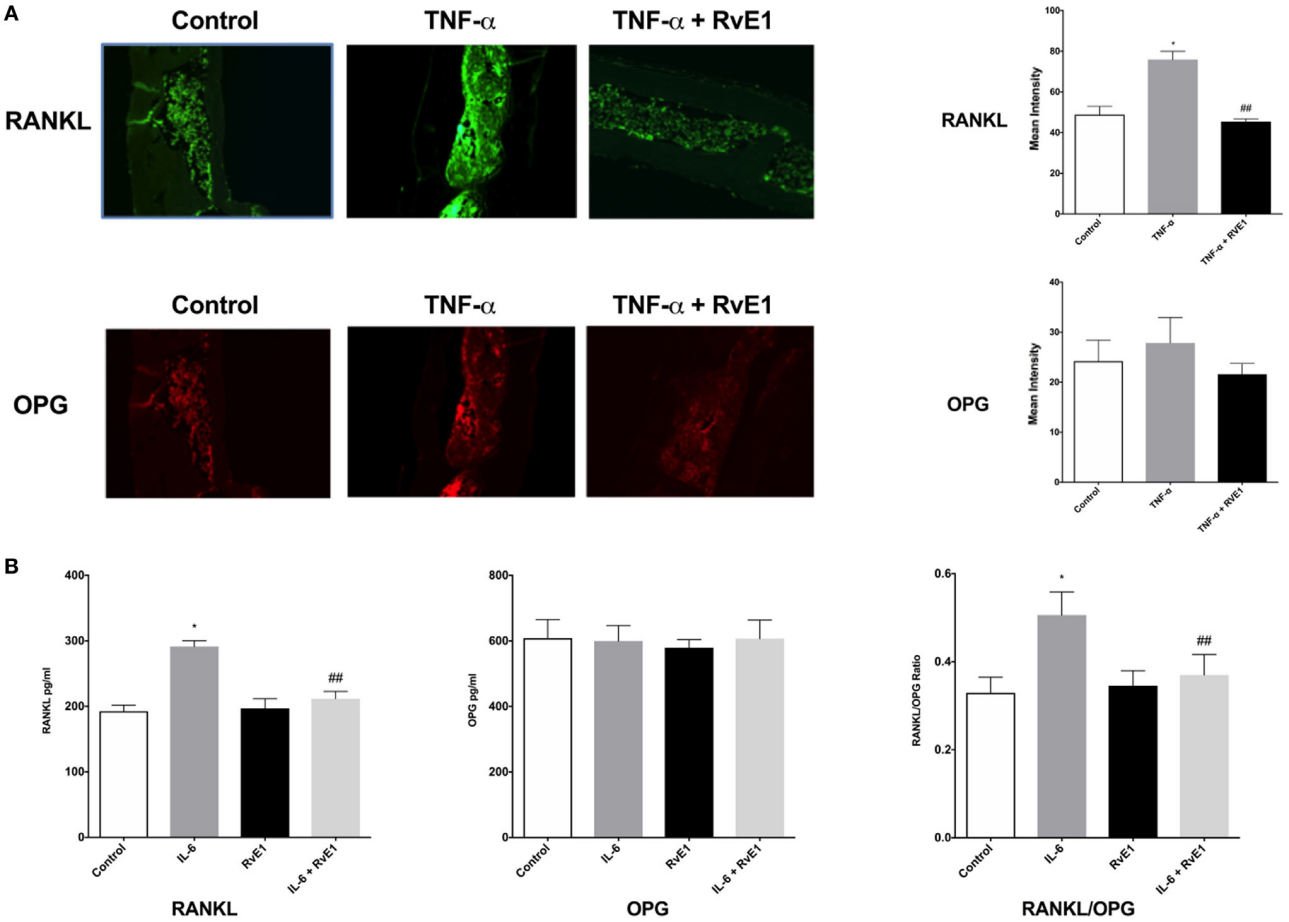
**(A)** Receptor activator of NFκB ligand (RANKL) and osteoprotegerin (OPG) balance is rescued by resolvin E1 (RvE1). Representative immunofluorescence imaging of primary RANKL monoclonal mouse antibodies conjugated with Alexa Fluor488. Evaluation of Immunofluorescence of histology sections in the different groups was conducted by taking 20× and 40× images and using Image J software to compare intensity of fluorescence in medullary areas. The mean immunofluorescence levels were then calculated and compared across groups. In the RvE1 treatment group, mean RANKL immunofluorescence was lower than the tumor necrosis factor-α (TNF-α) group (*p* < 0.05). The same slides stained with Alexa Fluor488 for RANKL immunofluorescence were also double stained with primary OPG monoclonal mouse antibodies conjugated with Dylight550 dye. Evaluation of mean OPG immunofluorescence values revealed no statistically significant differences between all three groups (*p* > 0.05). **(B)** Secreted RANKL and OPG are influenced by RvE1. The results demonstrate that RvE1 suppresses levels of RANKL *via* direct action on osteoblasts leading to a shift in the RANKL/OPG ratio in favor of bone preservation and formation. RvE1 at 100 nM significantly decreased RANKL production by osteoblasts stimulated by interleukin (IL)-6 (*p* < 0.02). Results are expressed as mean of RANKL and OPG levels. *p < 0.05 vs. control. ^##^*p* < 0.05 vs. active control.

The same sections from each group were double stained with Alexa Fluor 488 for RANKL and OPG monoclonal mouse antibodies conjugated with Dylight 550 dye (Figure 2A). In the RvE1 treatment group, mean RANKL immunofluorescence was lower than the TNF-α group. This difference was statistically significant (*p* < 0.05). Evaluation of mean OPG immunofluorescence values revealed no statistically significant differences between the three groups (*p* > 0.05).

### RvE1 Modulates RANKL Expression in Osteoblasts

Resolvin E1 at 100 nM significantly decreased RANKL production by osteoblasts stimulated by IL-6 (*p* < 0.05) (Figure 2B). RvE1 had no statistically significant effect on OPG production. RvE1 had also no effect on OPG and RANKL in the absence of IL-6. However, in the presence of IL-6, RANKL levels significantly increased and were reduced back to control levels after RvE1 at 100 nM was added (*p* < 0.05). Comparing the RANKL/OPG ratio between groups revealed a significant increase after the addition of IL-6 compared to negative controls. This increase was statistically significant (*p* < 0.05). Treatment with RvE1 at 100 nM led to a significant reduction in the RANKL/OPG ratio (*p* < 0.05). No significant difference was found between the groups with cells alone, when compared to cells with RvE1 alone. The results indicated that RvE1 suppresses levels of RANKL *via* direct action on osteoblasts leading to a shift in the RANKL/OPG ratio in favor of bone preservation and formation.

### RvE1 Differentially Regulates Gene Expression Under Inflammatory Conditions

**Figure 3:**
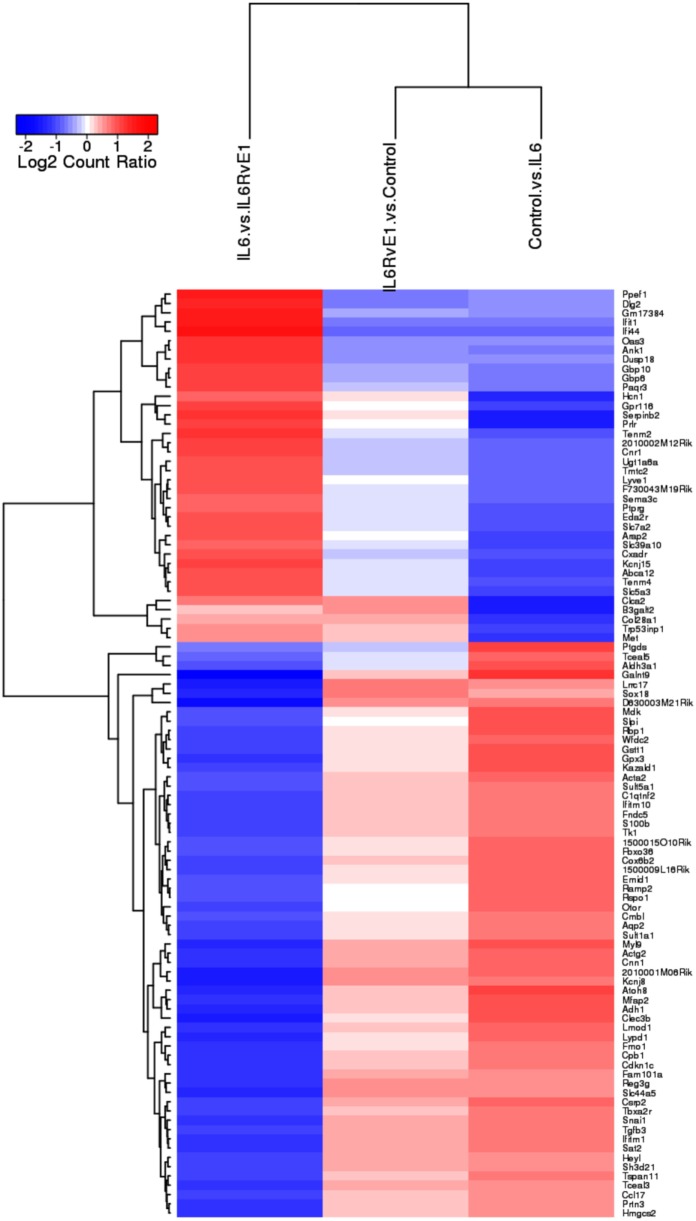
Gene expression shift with resolvin E1 (RvE1) treatment. A heat map of top 100 differentially expressed genes (absolute log_2_ fold change) compares OB + interleukin (IL)-6 vs. OB alone, OB + IL-6 + RvE1 vs. OB alone, and OB + IL-6 + RvE1 vs. cells + IL-6. The color intensity reflects the level of log_2_ fold change for each comparison with red for upregulation and blue for downregulation. Fainter colors indicate lesser changes in gene expression between the groups being compared. Genes were ordered based on a dendrogram derived from hierarchical clustering of log_2_ values of all rows; genes with similar differential expression patterns were grouped together. The result demonstrates a remarkable mirror image change in gene expression. Results are expressed as mean of genes highly expressed.

A heat map (Figure 3) demonstrating the top 100 differentially expressed genes (absolute log_2_ fold change) under different experimental conditions was constructed. The result demonstrated a remarkable mirror image change in gene expression in cells under inflammatory conditions and after RvE1 treatment. After RvE1 treatment, genes that were downregulated by the addition of IL6 were upregulated. Similarly, genes that were upregulated by IL-6 were downregulated after RvE1 treatment.

**Figure 4:**
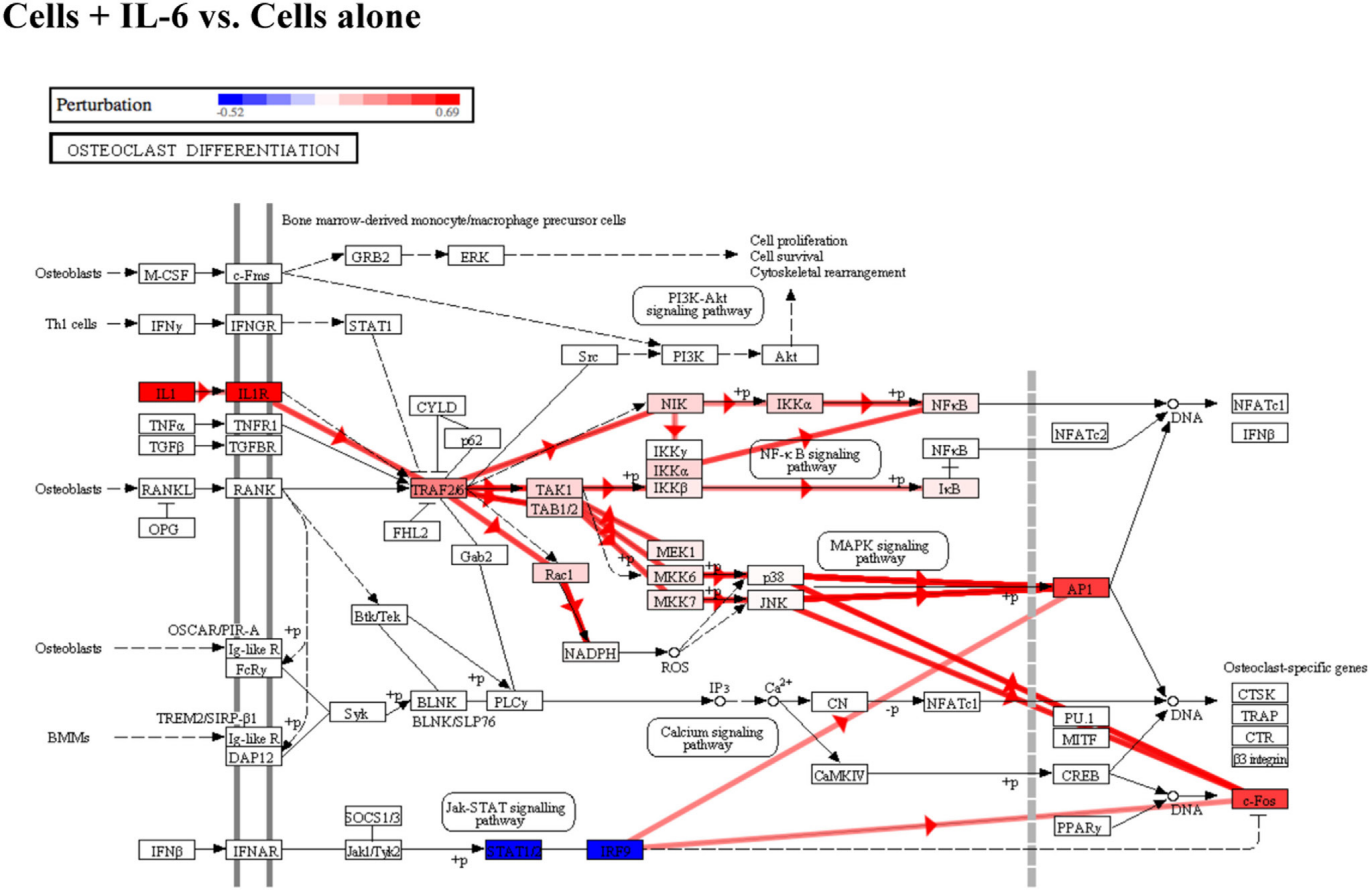

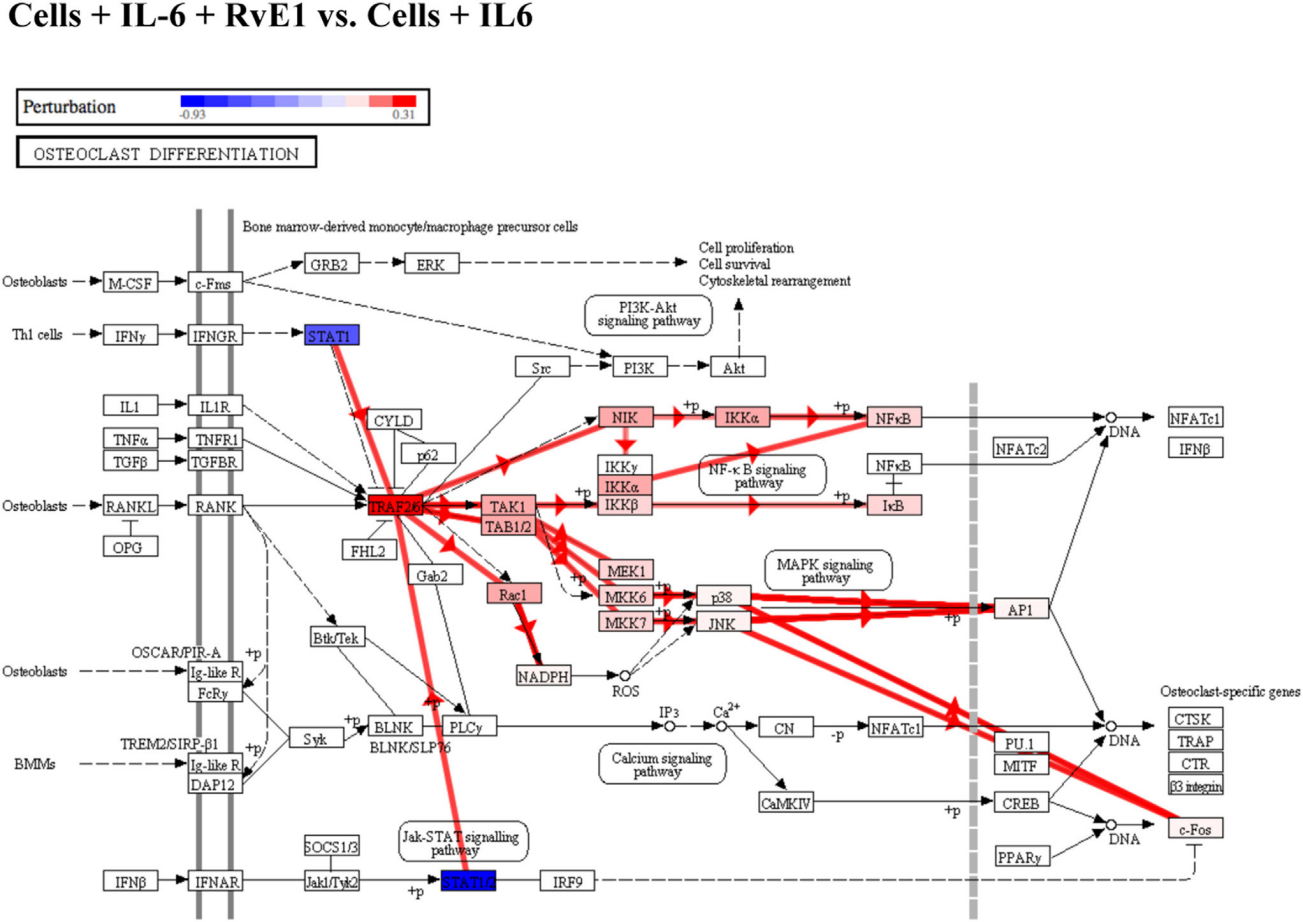
Osteoclast differentiation pathway perturbation after stimulation with interleukin (IL)-6 with or without resolvin E1 (RvE1). Using Illumina’s iPathwayGuide application, a significant perturbation in the osteoclast differentiation pathway was noted after stimulation of osteoblasts with IL-6. Upregulation of IL-1 lead to promotion of downstream signaling affecting the MAPK and NFκB signaling pathways leading to upregulation of osteoclast differentiation. After treatment with RvE1, downregulation of STAT1 led to inhibition of downstream signaling affecting the MAPK and NFκB signaling pathways leading to downregulation of osteoclast differentiation.

We then examined osteoclast differentiation pathway perturbation (Figure 4) after stimulation with IL-6 with or without 100 nM of RvE1. IL-6 caused an upregulation of IL-1 promoting a series of downstream signals affecting the MAPK and NFκB signaling pathways leading to upregulation of osteoclast differentiation. The addition of RvE1 led to downregulation of STAT1 and downstream signaling leading to suppression of osteoclast differentiation.

**Figure 5:**
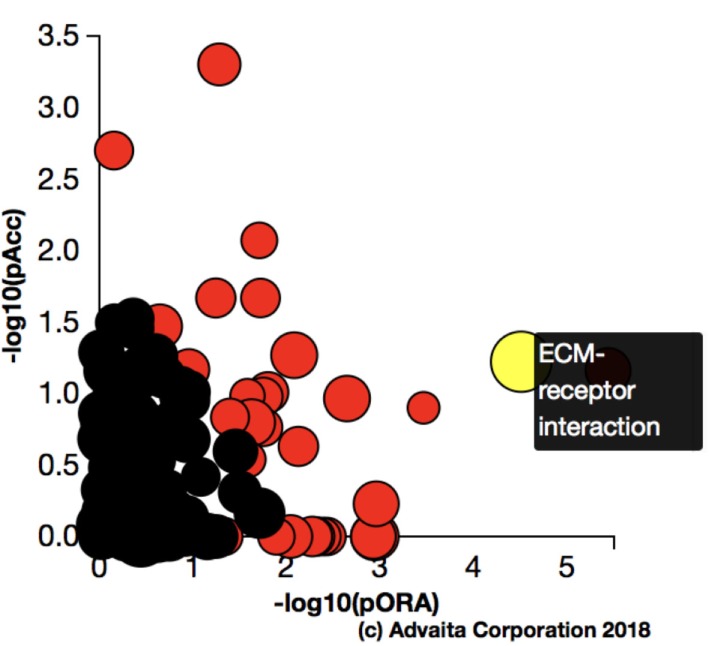
Pathway impact analysis. Using Illumina’s iPathwayGuide, we identified the biological pathways with the most significant perturbation and overrepresentation combination. Extracellular matrix (ECM) receptors interactions pathway was the most significantly affected.

**Figure 6:**
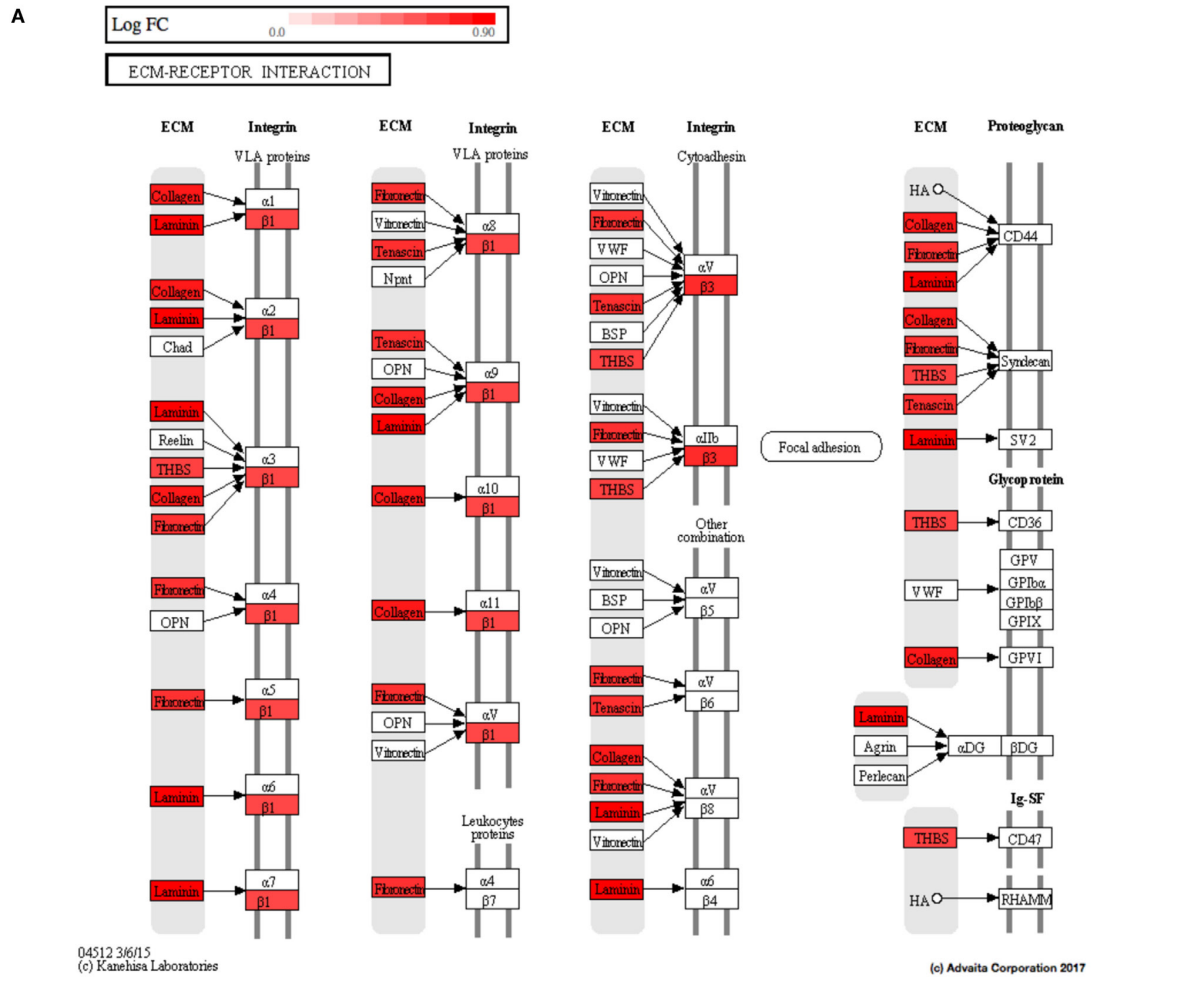

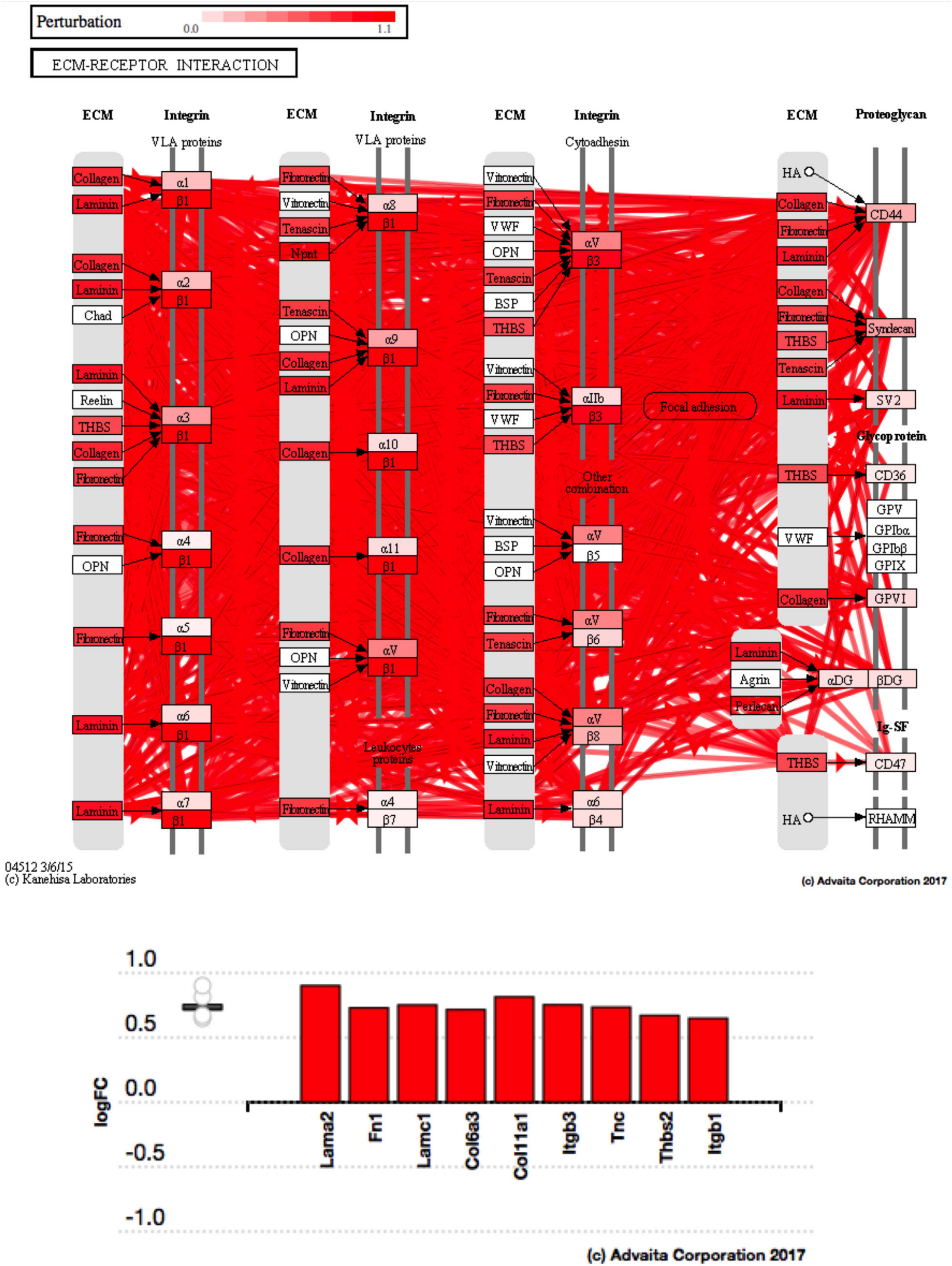

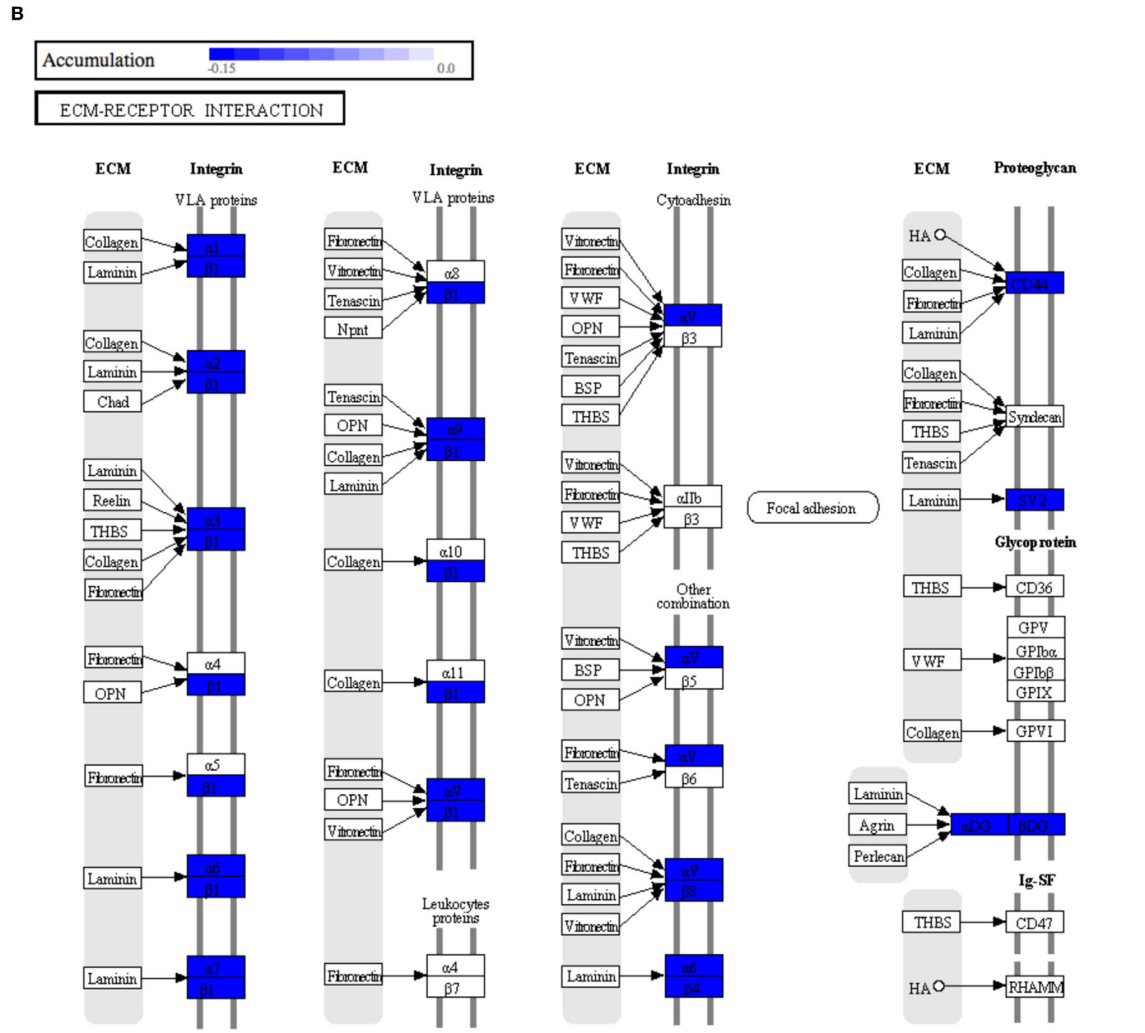

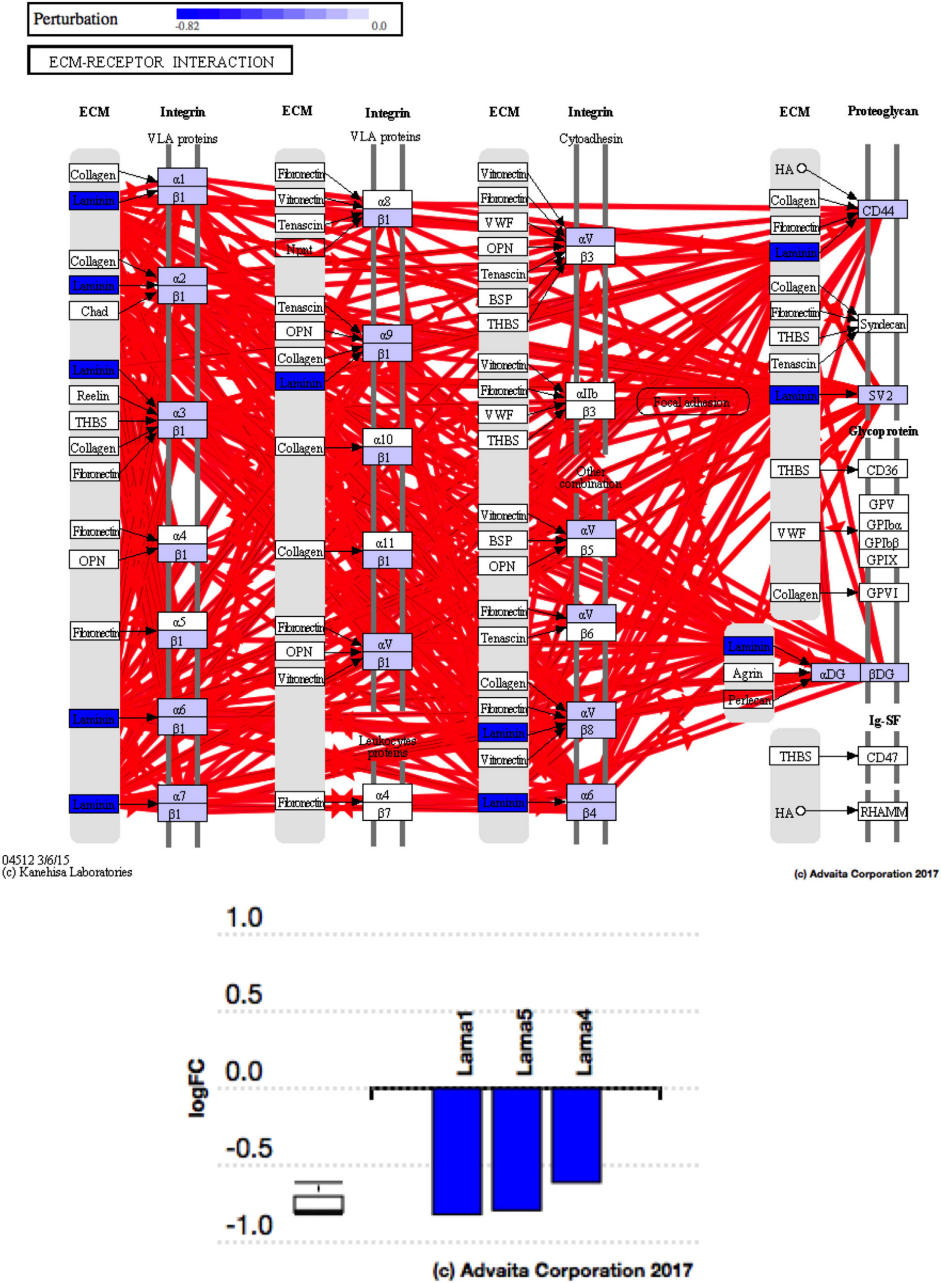
**(A)** Perturbation in extracellular matrix (ECM) receptor interactions in response to stimulation of osteoblasts with interleukin (IL)-6. Using Illumina’s iPathwayGuide, we evaluated the perturbation in ECM receptor interactions. Under inflammatory conditions, expression of ECM receptors aids in inflammatory cellular migration and adhesion. The diagram demonstrates a significant upregulation of ECM receptor interactions in response to stimulation of osteoblasts with IL-6. **(B)** Perturbation in ECM receptor interactions in response to resolvin E1 (RvE1) treatment after stimulation of osteoblasts with IL-6. The diagram demonstrates a significant downregulation of ECM receptor interactions in response to 100 nM RvE1 treatment after stimulation of osteoblasts with IL-6.

**Figure 7:**
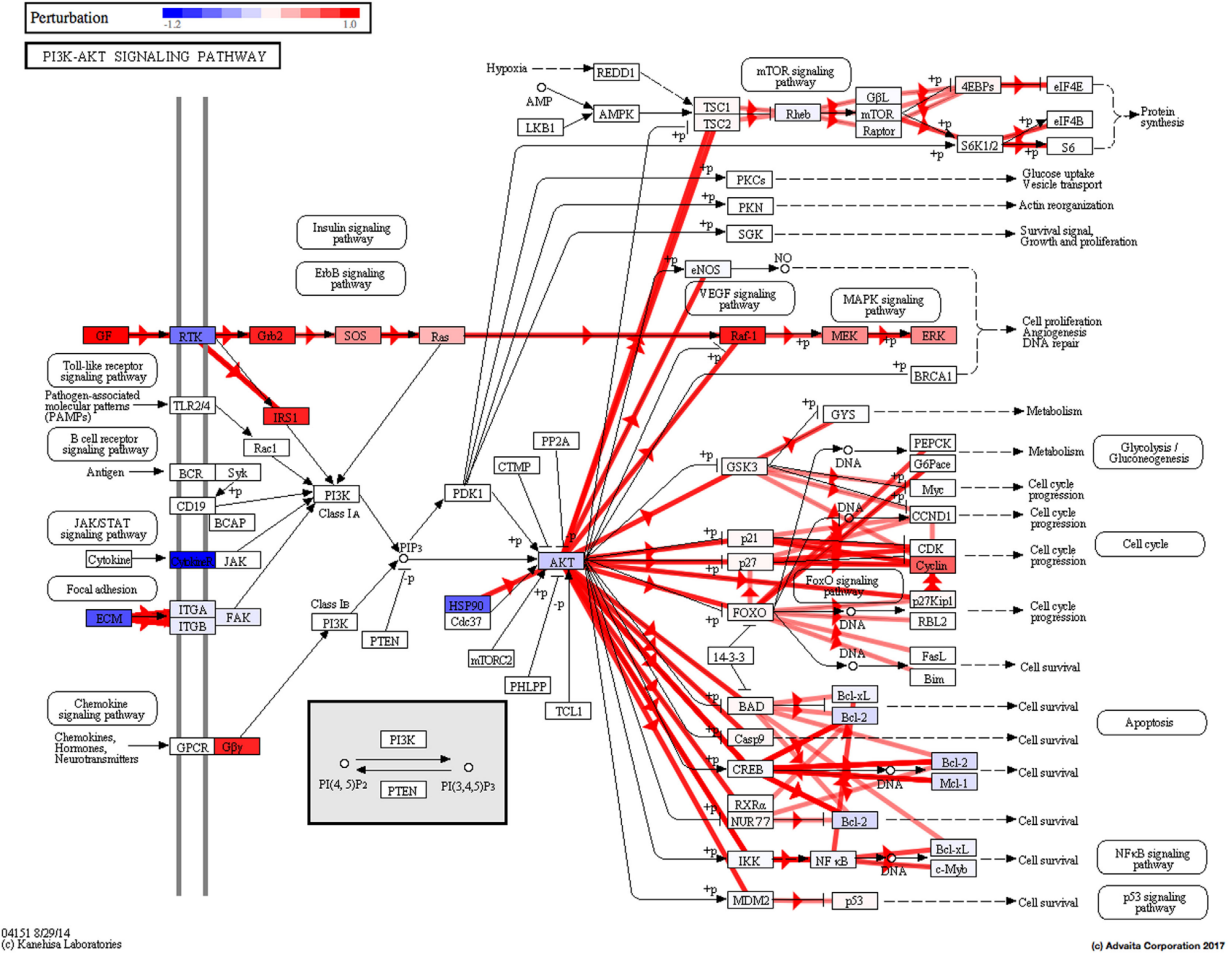
PI3K–AKT pathway perturbation in response to treatment of interleukin (IL)-6 stimulated osteoblasts with resolvin E1 (RvE1). The perturbation demonstrates that RvE1 influences PI3K–AKT by regulating multiple upstream and downstream targets and thereby affecting multiple cell functions including differentiation, protein synthesis, and apoptosis. These interactions also influence the NFκB, MAPK, and p53 signaling pathways.

**Figure 8:**
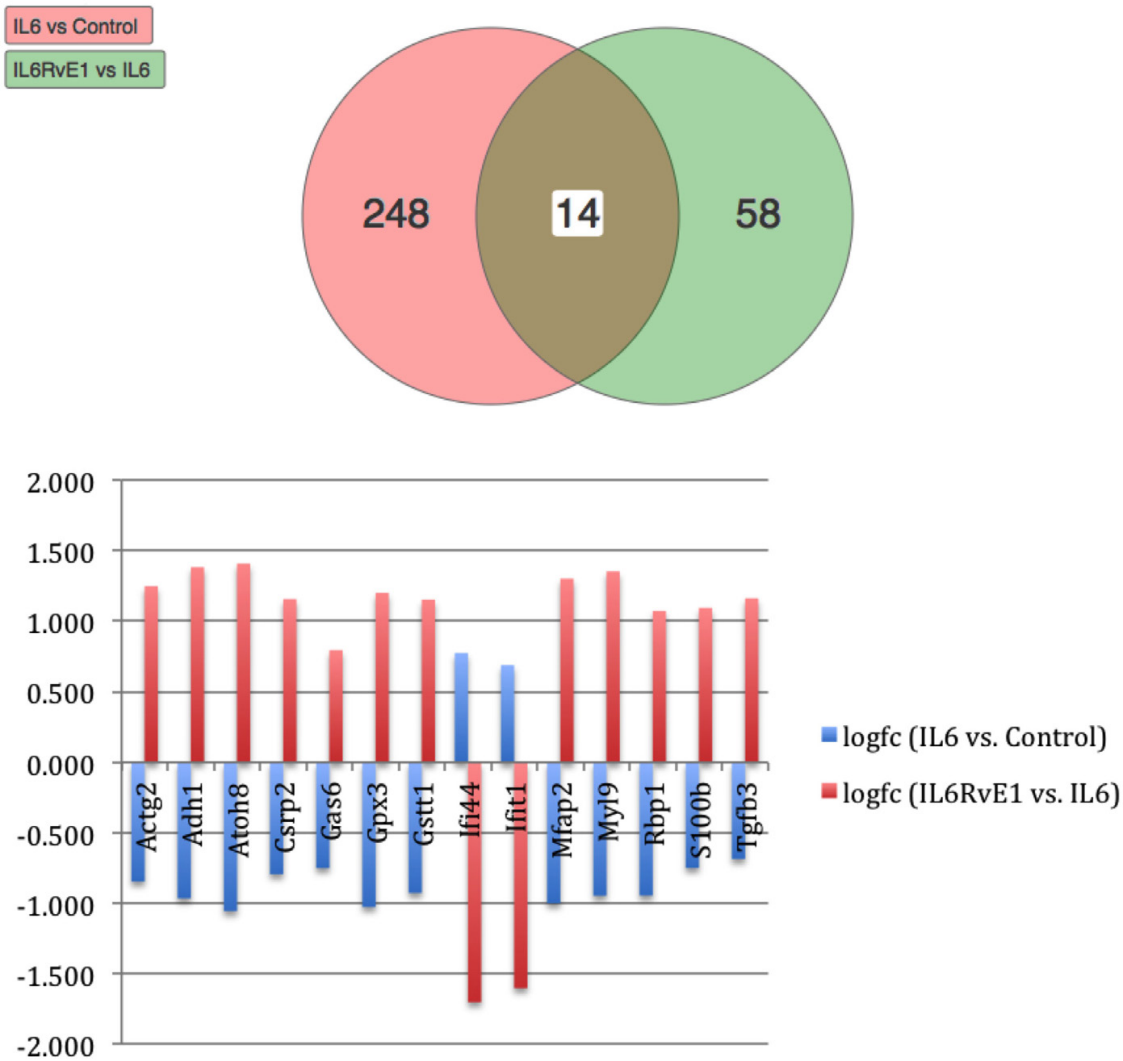
Meta analysis of differential gene expression. We identified a group of 14, which were significantly differentiated (upregulated/downregulated) genes after induction of inflammation and were significantly reversed after treatment with resolvin E1 (RvE1). For a list of genes and their functions see Tables 1 and 2.

Using Illumina’s iPathway Guide impact analysis tool (43, 44), we identified the biological pathways with the most significant perturbation and overrepresentation combination (Figure 5). The extracellular matrix (ECM) receptor interactions were found to be the most significant. Under inflammatory conditions, expression of ECM receptors aids in inflammatory cellular migration and adhesion. After stimulation with IL-6, osteoblasts demonstrate a significant upregulation of ECM receptors (Figure 6A). This upregulation leads to a high fold change in the level of ECM receptor interaction perturbation. After treatment with 100 nM of RvE1, a significant downregulation of the ECM interactions is noted, which promoted a high-fold downregulation in the level of ECM receptor interaction perturbation (Figure 6B). The PI3K–AKT pathway also showed a significant perturbation in response to treatment of IL-6 stimulated osteoblasts with RvE1 (Figure 7). Treatment with RvE1 significantly impacted the PI3K–AKT by regulating multiple upstream and downstream targets and thereby affecting multiple cell functions including differentiation, protein synthesis, and apoptosis. These interactions also influence the NFκB, MAPK, and p53 signaling pathways. We further conducted a meta-analysis on differential gene expression under the three experimental conditions, comparing cells + IL-6 vs. cells alone, IL-6 + RvE1 vs. cells alone and cells + IL-6 + RvE1 vs. cells + IL-6. We isolated 14 genes that were significantly differentially regulated after stimulation with IL-6 whose regulation was significantly reversed by RvE1 treatment (Tables 1 and 2; Figure 8).

**Table 1:** List of genes differentially regulated by RvE1 treatment.

Gene	logfc		logfc	

	[interleukin (IL)-6 vs. control]	*p* Value	(IL6RvE1 vs. IL6)	*p* Value
Actg2	−0.844	0.006	1.248	0.043
Adh1	−0.964	0.008	1.383	0.000
Atoh8	−1.054	0.000	1.408	0.000
Csrp2	−0.793	0.002	1.156	0.046
Gas6	−0.749	0.010	0.794	0.040
Gpx3	−1.025	0.000	1.200	0.002
Gstt1	−0.925	0.011	1.153	0.041
Ifi44	0.774	0.001	−1.700	0.000
Ifit1	0.688	0.044	−1.601	0.000
Mfap2	−0.998	0.000	1.302	0.003
Myl9	−0.945	0.006	1.353	0.023
Rbp1	−0.944	0.000	1.073	0.045
S100b	−0.748	0.030	1.094	0.002
Tgfb3	−0.684	0.040	1.162	0.014

**Table 2:** Actions of genes significantly regulated by RvE1 treatment.

Gene symbol	ID	Title	Summary of actions
Actg2	25365	Actin, gamma 2, smooth muscle, enteric	May play a role in smooth muscle function
Adh1	24172	Alcohol dehydrogenase 1	Alpha subunit of class I alcohol dehydrogenase; metabolizes a wide variety of substrates including ethanol, hydroxysteroids, and lipid peroxidation products
Atoh8	500200	Atonal bHLH transcription factor 8	Regulates skeletal myogenesis
Csrp2	29317	Cysteine and glycine-rich protein 2	May act as an adaptor molecule in the JAK/STAT signaling pathway
Gas6	58935	Growth arrest specific 6	Provides protection of neurons against serum deprivation-induced apoptosis
Gpx3	64317	Glutathione peroxidase 3	The protein encoded by this gene belongs to the glutathione peroxidase family, members of which catalyzes the reduction of organic hydroperoxides and hydrogen peroxide (H_2_O_2_) by glutathione, and thereby protect cells against oxidative damage
Gstt1	25260	Glutathione S-transferase theta 1	Subunit of the glutathione S-transferase
Ifi44	310969	Interferon-induced protein 44	Unknown
Ifit1	56824	Interferon-induced protein with tetratricopeptide repeats 1	Responses to dexamethasone and other inflammatory stimuli
Mfap2	313662	Microfibril associated protein 2	Unknown
Myl9	296313	Myosin light chain 9	Unknown
Rbp1	25056	Retinol binding protein 1	Binds and transports retinol; plays a role in vitamin A metabolism
S100b	25742	S100 calcium binding protein B	Binds GTPase activating protein IQGAP1; may play a role in cell membrane rearrangement
Tgfb3	25717	Transforming growth factor, beta 3	Involved in epithelial and endothelial cell proliferation and differentiation during development

## Discussion

Inflammation and bone remodeling are inseparable processes; however, little is known about the details of interaction between the immune response and bone. Clearly, certain inflammatory cytokines are known to have direct actions on bone (IL-1 and IL-6), but the cellular mechanisms that regulate these actions are poorly understood. It has been demonstrated that in inflammatory diseases the RANKL/OPG ratio is consistently elevated (46, 47). RANKL is the primary signal for osteoclastic differentiation, while OPG acts as a decoy receptor that inhibits RANKL–RANK binding and thus decreases osteoclastogenesis. Therefore, the RANKL/OPG ratio drives osteoclastogenesis and osteoclast activation (48).

To evaluate whether the regulation of the RANKL/OPG ratio by RvE1 is partially due to direct actions on bone cells or not, primary neonatal osteoblasts were isolated from calvariae of WT mice. Neonatal mouse osteoblasts, in response to inflammation, have been shown to stimulate the release of RANKL (49) and inhibit secretion of its decoy receptor OPG (50), promoting osteoclastogenesis and bone resorption (51). Two separate research groups have independently demonstrated that osteoblasts express chemR23 receptor (52, 53). Our *in vitro* finding that RvE1 acts directly on osteoblasts to decrease the RANKL levels, elevated under inflammatory conditions is, to the best of our knowledge, a novel finding. These observations confirmed earlier findings that RvE1 regulates the RANKL–OPG ratio to favor bone preservation (53). It also provides a clarification that RvE1 treatment of bone marrow-derived primary osteoclast cultures results in a decrease of osteoclastic maturation and thereby decreased bone resorption *in vitro* (54).

Animal and human trials have demonstrated that regular diets rich in ω-3 PUFA result in a reduction in bone turnover and an increase bone mineral density (55–58). However, the exact mechanism of action of ω-3 PUFA on a molecular level is still not completely clear. In bone marrow samples harvested from rats fed a diet supplemented with EPA, elevated levels of RvE1 have been noted (59). Our findings demonstrate that RvE1, the active metabolite of EPA, impacts bone remodeling under inflammatory conditions and that this impact is, in part, due to a direct action on bone cells. Our RNA-Seq and differential expression analysis data enabled us to understand more about the receptors and signaling pathways through which RvE1 directly acts on osteoblasts. RvE1 differentially regulated gene expression in response to inflammation and reversed gene expression levels back to homeostasis levels. We also isolated pathways with significant overrepresentation and perturbation in response to IL-6 stimulation with or without RvE1. The osteoclast differentiation pathway showed perturbation after stimulation with IL-6 with or without 100 nM of RvE1. Our perturbation analysis revealed that this might be partially due to a sharp upregulation of IL-1 expression, which promoted a series of downstream signaling events affecting the MAPK and NFκB signaling pathways promoting upregulation of osteoclast differentiation. After the addition of RvE1, a downregulation of STAT1 led to downregulation of same downstream signaling pathways affected by IL-6 through IL-1, in both the MAPK and NFκB pathways. This highlighted the need to look at the broader actions of pro-resolution molecules and how they regulate different pathways through collateral signaling. We identified that the ECM receptor interaction pathway showed significant perturbation and over representation in response to IL-6 and after treatment with RvE1. ECM receptor interactions were sharply upregulated after stimulation with IL-6. This finding is consistent with our current knowledge of the role played by ECM in modulating inflammation (60). In addition to acting as a scaffold or barrier for cells infiltrating inflamed tissues, ECM regulates inflammatory cell migration through endothelial basement membranes, provides specific signaling to immune cells to identify their exit sites in venules, as well as regulating their ability to promote the inflammatory response (60). Treatment with RvE1 appears to have reversed the upregulation of ECM receptor interactions induced by IL6 suggesting a return to homeostasis. The PI3K–AKT pathway showed a significant perturbation in response to treatment of IL-6 stimulated osteoblasts with RvE1 (Figure 7). RvE1 acted on multiple upstream and downstream targets of AKT affecting multiple cell functions including differentiation, protein synthesis, and apoptosis. These interactions also influenced the NFκB, MAPK, and p53 signaling pathways. These findings confirm results by Ohira et al. showing that RvE1 stimulated phosphorylation of AKT that was both ligand and receptor dependent (61). They also are consistent with Berg et al.’s findings, that stimulation of chemR23 with the agonist chemerin resulted in downstream Akt and MAPK phosphorylation, as well as elevated levels of pro-inflammatory cytokines (62). The downregulation of Gas6 after stimulation with IL-6 and its upregulation after treatment with RvE1 is consistent with reported patterns of Gas6 expression. Gas6 has been shown to decrease the production of pro-inflammatory cytokines (63).

Chemokine like receptor 1 (ChemR23 or CMKL1) receptors for RvE1 have been identified on monocytes (64). These molecules also serve as receptors for chemerin (65, 66). Moreover, ChemR23 was found to be expressed in other tissues, such as renal, cerebral, gastrointestinal, and cardiovascular tissues (52, 67). Its inhibition of TNF-α stimulated NFκB activation seems to be directly related to its ability to specifically bind RvE1 (30). The specific mechanism by which pro-resolution molecules, downregulate bone loss during the course of inflammatory diseases is partially known (53), but how resolvins, specifically RvE1, directly impact bone cells is still poorly understood. The finding that ChemR23 is expressed in developing bone tissue might indicate a role in bone formation (68).

Together, our results provide evidence for RvE1’s direct impact on the skeletal system; regulating pathologic inflammation-induced bone resorption by control of the RANKL/OPG ratio and downstream genetic events.

## Ethics Statement

This study was carried out in accordance with the recommendations of “Institutional Animal Care and Use guidelines at Forsyth Institute.” The protocol was approved by the “Institutional Animal Care and Use Committee at the Forsyth institute.”

## Author Contributions

KEK and TVD contributed conception and design of the study. KEK performed all the experiments and wrote the first draft of the manuscript. TC performed transcriptomics and statistical analysis. MF did the analysis for MicroCT examination. KEK, TVD, and MF wrote sections of the manuscript. All authors contributed to manuscript revision, read and approved the submitted version. The corresponding author takes primary responsibility for communication with the journal and editorial office during the submission process, throughout peer review and during publication. The corresponding author is also responsible for ensuring that the submission adheres to all journal requirements including, but not exclusive to, details of authorship, study ethics and ethics approval, clinical trial registration documents, and conflict of interest declaration. The corresponding author should also be available post-publication to respond to any queries or critiques.

## Conflict of Interest Statement

The authors declare that the research was conducted in the absence of any commercial or financial relationships that could be construed as a potential conflict of interest.
